# Regeneration of Denervated Skeletal Muscles – Brunelli’s CNS-PNS Paradigm

**DOI:** 10.25122/jml-2019-0063

**Published:** 2019

**Authors:** Tobias von Wild, Giorgio A. Brunelli, Klaus R.H. von Wild, Marlene Löhnhardt, Cornel Catoi, Adriana Florinela Catoi, Johannes C. Vester, Stefan Strilciuc, Peter Trillenberg

**Affiliations:** 1. Department of Plastic Reconstructive and Aesthetic Surgery, Hand Surgery, Praxisklinik in der Alster City, Hamburg, Germany; 2. School of Specialists in Orthopedics, Traumatology, Hand and Microsurgery, University of Brescia, Brescia, Italy; 3. Foundation Giorgio Brunelli for Research on Spinal Cord Lesions ONLUS, E.S.C.R.I., Brescia, Italy; 4. Department of Neurosurgery, Medical Faculty Westphalia Wilhelm’s University Münster, Münster, Germany; 5. International Neuroscience Institute, Hanover, Germany; 6. Department of Plastic and Reconstructive Surgery, Hand Surgery, University Hospital, Hamburg, Germany; 7. Department of Pathology, University of Agricultural Science and Veterinary Medicine, Cluj-Napoca, Romania; 8. Department of Functional Biosciences, University of Medicine and Pharmacy “Iuliu Hatieganu”, Cluj-Napoca, Romania; 9. Department of Biometry & Clinical Research, idv Data Analysis and Study Planning, Gauting, Germany; 10. Department of Neurology, University of Medicine and Pharmacy “Iuliu Hatieganu”, Cluj-Napoca; Romania; 11. Department of Neurology, University Medical Center Schleswig-Holstein, Lübeck, Germany

**Keywords:** CNS – PNS graft, Brunelli’s paradigm, Muscle regeneration, Cerebrolysin neuromodulation, Traumatic paraplegia, ACh – acetylcholine, AChGL-CN – Cholinergic/glutamatergic co-neurotransmission, CC – Cornel Catoi, CERE – Cerebrolysin, CI – confidence interval, CMAP – compound muscle action potential, CST – cortical spinal tract, DM – Dafin F. Muresanu, EMG – electromyogram, GB – Giorgio Brunelli, HPF – high-power fields, HRQOL – Health-related quality of life, ICU – intensive care unit, IOAM – internal oblique abdominal muscle, IP – intraperitoneal, IV – intravenous, KvW – Klaus RH von Wild, ML – Marlene Löhnhardt, NEZ – nerve entrance zone, NG – nerve graft, NMJ – neuromuscular junction, NR – neurorehabilitation, PNG – Peripheral Nerve Graft, RCT – randomized controlled trial, SC – spinal cord, SCI – spinal cord injury, SNG – sural nerve graft, TvW – Tobias von Wild

## Abstract

The restoration of voluntary muscle activity in posttraumatic paraplegia in both animal experiments and other clinical applications requires reproducibility of a technically-demanding microsurgical procedure, limited by physicians’ understanding of Brunelli’s spinal cord grafting paradigm. The insufficient clinical investigation of the long-term benefits of the CNS-PNS graft application warrants additional inquiry.

The objective of this study is to explore the potential benefits of the first replicated, graft-induced neuroregeneration of denervated skeletal muscle regarding long-term clinical outcomes and to investigate the effect of Cerebrolysin on neuromodulation.

A randomized study evaluating 30 rats, approved by the National Animal Ethics Advisory Committee was performed. The medication was administered postoperatively. For 14 days, 12 rats received Cerebrolysin (serum), 11 received NaCl 0.9% (shams), and 7 were controls. For microsurgery, the lateral corticospinal tract T10 was grafted to the denervated internal obliquus abdominal muscle. On day 90, intraoperative proof of reinnervation was observed. On day 100, 15 rats were euthanized for fixation, organ removal, and extensive histology-morphology examination, and the Wei-Lachin statistical procedure was employed.

After an open revision of 16 rats, 8 were CMAP positive. After intravenous Vecuronium application, two (Cerebrolysin, NaCl) out of two rats showed an incomplete compound muscle action potential (CMAP) loss due to glutamatergic and cholinergic co-transmission, while two others showed a complete loss of amplitude.

Cerebrolysin medication initiated larger restored muscle fiber diameters and less scarring. FB+ neurons were not observed in the brain but were observed in the Rexed laminae.

Brunelli’s concept was successfully replicated, demonstrating the first graft induced existence of cholinergic and glutamatergic neurotransmission in denervated grafted muscles. Statistics of the histometric count of muscle fibers revealed larger fiber diameters after Cerebrolysin.

Brunelli’s CNS-PNS experimental concept is suitable to analyze graft-neuroplasticity focused on the voluntary restoration of denervated skeletal muscles in spinal cord injury. Neuroprotection by Cerebrolysin is demonstrated.

## Introduction

In 1981, Giorgio Brunelli (GB) began his experimental research in a rat model “to connect the cephalad cord (in which regrowing axons are present) to peripheral nerves (in which axon growth can advance) to overcome the non-permissiveness of the spinal cord after severance”. In 1994, he used his paradigm of direct neurotization of denervated skeletal muscle via presynaptic motoneurons in nonhuman primates (Macaca fascicularis) to finally demonstrate “new voluntary innervation of some hip muscles that after laminectomy T8 to T11 had been directly connected by sural nerve grafts from the lateral bundle of the spinal cord.” His ultimate target was “to search for a new microsurgical concept for functional restoration in human paraplegics that can provide human paraplegics at least a few elementary voluntary movements as to stand up and ambulate with the aid of walkers” [1]. Consequently, in 2000-2002, with the permission of the Ethical Committee of the Italian SSN, GB and KvW operated on three selected human spinal cord injured patients with paraplegia, performing this experimental concept of restorative surgery by CNS-PNS graft [2-4]. Only one of the operatively treated patients, lady G., completed her intensive mandatory postoperative physical rehabilitation at present. She became able, when continuing her daily exercises, to stand up, to walk with tetrapod sticks up to 100 m, and to climb steps at her home several times a day to enjoy a reasonable quality of life [4,5]. In 2015, she first agreed to undergo a functional magnetic resonance tomography (fMR) examination on GB’s special request, with whom she was in personal contact since her operation [6].

To date, a replication of GB’s concept for CNS-PNS graft either in animals or human restorative SCI surgery has not been reported elsewhere in the literature, as it was not recommended for clinical use [7-10,11]. GB’s paradigm of first motoneuron regeneration to rectorate denervated muscle function, therefore, warranted scientifically presented proof of muscle regeneration by external inquiry [1]. In 2008, after introduction by GB, the replication of GB’s paradigm in a rat model was performed by TvW at the Department of Plastic and Hand Surgery, under the direction of Peter Mailänder, MD, Prof., Medical Faculty UK-SH, Campus Lübeck, Germany; this replication was performed when all data regarding GB’s paradigm had been extensively analyzed by a European transdisciplinary expert team [11]. 

This second report is focused on the evidence of peripheral nerve graft (PNG) induced regeneration of denervated internal oblique abdominal muscle (IOAM) fibers, as the authors were ultimately interested in using GB’s paradigm for a new alternative microsurgical concept in restorative microsurgery of human brachial plexus and SCI lesions. Based on the literature reports on beneficial neuroprotective pharmacological neuromodulation, GB’s study design was extended as a prospective randomized, double-blinded, placebo-controlled trial (RCT) using Cerebrolysin® (CERE) medication postoperatively [11-18].

## Material and Methods

### Animal subjects

Secondary data analyses of evidence from our previously reported graft-induced neuroplasticity in rats after replicating Brunelli's CNS-PNS concept were included [1,2,11]. Brunelli's concept was extended to pharmacological neuromodulation by postoperative CERE medication in a transdisciplinary RTC double-blinded trial [12-18]. The surgery, caring, and housing of rats was performed at the veterinarian surgical labs in compliance with the National Research Guide for the Care and Use of Laboratory Animals and was ethically approved by the Animal Research Committee of the University of Schleswig, Holstein county, Germany, (Research number: V 312-72241.1). Governmental approval and control of legality was provided by the Christian-Albrecht-University, Kiel, German and Institute of Animal Breeding and Husbandry, in compliance with the European Commission Directive 2010/63/EU [19]. All efforts were taken to minimize animal suffering and the number of subjects included in the study [20-25]. Governmental approval was given for 30 adult white female Sprague Dawley rats (Charles River, 220 to 280 g). Experimental procedures included prospective RCS with simple preoperative randomization of three groups via coin-tossing by the veterinarian physician. The two treatment groups included CERE (serum) and NaCl (shams) by double-blinded IP application over 14 days as well as controls (nil) [12,13]. CERE, a peptidergic nootropic drug, is known for its pleiotropic neuroprotective effect and has a mechanism that can protect neurons from neurodegeneration induced axotomy [14-18]. This medication was dependent on the rats' preoperative physical and behavioral state on day 1 of the operation [19-25]. Members of the research team were kept blinded to medication (postoperative intraperitoneal injections) until all histology-morphology examinations were completed and reviewed, and the statistical analyses were described [26-40]. When the results were unblinded, the RCT distribution showed 12 CERE (serum), 11 NaCl (shams), and 7 controls (nil).

We previously provided a summary of all pharmaceuticals and biochemical products used, the applied dosages, routes of administration, and products in more detail [11]. For sedation, CO2 99% insufflation in combination with O2 30% was administered, and ether pro narcosis 50 ml drop-inhalation until sedation and sleep. General anesthesia during the 1st, 2nd, and 3rd operations was obtained by using IP Urostamin ®,100 ml Rampur® ad us. vet. and a Ketamine/Xylazine rat cocktail ad us. vet.; local intra and postoperative anesthesia were achieved using Scandicain® 5 ml 2% SC/IM [24,25]. Euthanasia was performed only under deep general anesthesia by intraventricular injection of KCl 1-2 /kg IV (third operation). Antibiotic treatment involved metamizole 500 mg, 20 ml drops postoperatively for ten days to alert animals; also, Doxycycline-Ratiopharm SF 100 mg/5 ml was administered at 10 mg/kg two times daily postoperatively for a duration of 6 days. Vecuronium at 10 mg/5 ml, 2,5 mg/ml IV application was administered for competitive ACh-neuromuscular blocking daily until day 14, and neuromodulation was achieved by Cerebrolysin 5 mg/kg single dose 25 ml IP postoperatively.

Frequent sudden deaths of unknown objective pathology that are known from the investigation of Brunelli's concept in animal research occur, but these deaths occur less frequently after the introduction of postoperative intensive care management. A fatal outcome is registered in the daily protocol, analyzed together by the veterinarian toxicologist, pharmacologist, and pathologist, and reported to the government department of animal research.

### Surgical procedures

Eleven materials and equipment used in restorative microsurgery were as follows: Leica Wild M-690 Surgical Microscope; Biomed atraumatic bipolar electro muscle and nerve-stimulator; Ethilon monofil 9-0, 10-0 (not absorbable), Vicryl 3-0 (absorbable); maniple wound closure staple gun AZ 35 W 35 B 6,9 mm, H 3.6; mechanical animal ventilator (Pmax 18 mbar, ventilation frequency – 50/min, flow – 1.5 l/min, inspired oxygen fraction (FiO2) – 0.7); intra and postoperative warm circulating water blanket to prevent hypothermia and to maintain the rats' body temperature within the physiological range [20-22,24,25].

**Figure 1:**
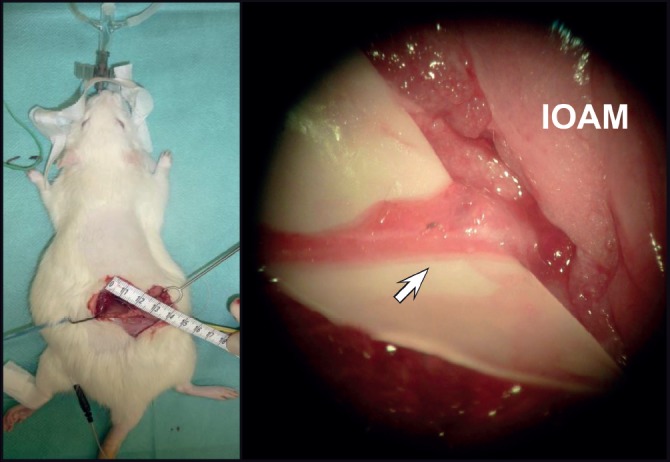
CNS-PNS grafting from right lateral CST T10 to NEZ of the IOAM (first operation). Operation situs: Microsurgical identification of the IOAM and its nerve entry zone (NEZ).

**Figure 2:**
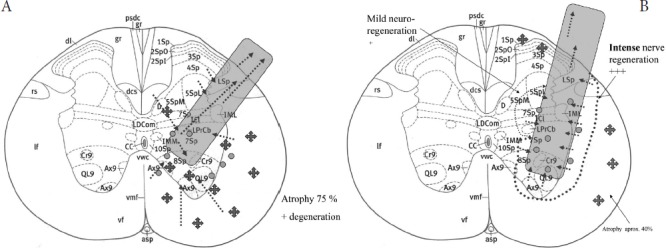
AB. PNG (CAMP+) at histology-morphology Rexed areas T10. Black dark cross signs - scar formation, atrophy; grayish dots and arrows - axonal regeneration and sprouting next to the graft, within the SC and the graft. Explanation: The Rexed laminae of spinal gray matter, medial (M) or lateral (L) part, axial (Ax) or abaxial (Ab); D - Dorsal nucleus (Clarke); dc -dorsal columns; dcs – dorsal. SNG position (gray shapes); black cross signs indicate histological-morphological intensity of atrophy/degeneration; dotted black arrows show axonal regeneration and spouting; area of small grayish dots and dotted black arrows indicate axonal sprouting and regeneration, tiny dotted lines circumscribe the regions and intensity of graft-induced neuroregeneration; (left): morphology specimen No. 5 (no medication) Rexed areas T10, lateral CST; moderate axonal regeneration of SC and PNG; 75% atrophy/degeneration, fibrosis.

First operation: CNS-PNS graft procedure in the prone position, four steps (Figure 1) [21-23]:

1. Harvesting of the right sural nerve graft (SNG);
2. Skin incision, medial longitudinal 50 mm, microsurgical preparation and electrical identification of IOAM – NEZ (Figure 2);
3. Laminectomy from T10 to L1;
4. At T10, incision of the dura mater and arachnoid membrane was performed dorsally (mediolateral on the right side); preparation of the right SC dorsolateral bundle for the free-hand stitch incision (1.5 to 2 mm deep) directed toward the lateral cortical spinal tract and introduction of the proximal PNG end, fixed at the arachnoid membrane by one stitch 9-0 atraumatic; end to end adaptation of the graft end to the cut distal IOAM nerve stump, next to its NEZ, fixed by one atraumatic stitch 9-0. After hemostasis and wound closure, blinded medication was injected intraperitoneally in the supine position before ICU observation.

Second operation at day 90: Open microsurgical check of graft's intact extradural position and IOAM nerve adaptation; CAMP assessment of neurodegeneration [26,27]. Depending on the individual positive CAMP result, proof of the neurotransmitter type obtained either by central first and/or peripheral second motoneuron as demonstrated by IV application of Vecuronium during artificial ventilation (oropharyngeal intubation) was assessed. Finally, Fast Blue is directly applied around the IOAM nerve fascicles at NRZ to indicate graft-induced retrograde axonal staining within the graft, PNS, and CNS.

**Figure 3:**
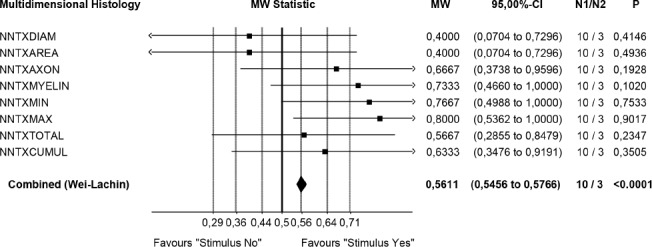
Statistical evidence of graft induced IOAM fiber diameters (ML, Lübeck) * Restricted precision of test procedures due to low sample size Wei-Lachin Procedure (multidimensional histology), stimulus “Yes” vs. stimulus “No” effect sizes and two- sided 95% C.I.: Significant superiority of the “stimulus yes” group versus the “stimulus no” group (MW = 0.5611, 95%-CI 0.5456 – 0.5766) demonstrated by histometric evaluation of grafted muscle fibers diameters. Validated evidence that bigger muscle fibers correlate with regenerated/restored functional CAMP+

Third [11] operation at day 100: We performed euthanasia to harvest the total brain, SC, NG, and both IOAMs for distinct histology-morphology examinations and histometric measuring of graft-induced changes, e.g., axonal regeneration, cell death and scarring, polyfocal regeneration, and others [31-43]. In this report, we focus on the regeneration of denervated grafted muscle fiber diameters compared with intact controls in Lübeck (ML) and Cluj (CC), both with and without CERE medication. Electrophysiological diagnostics (EMG, CMAP) [26,27]: CMAP idealizes the summation of a group of almost simultaneous action potentials from several muscle fibers in the same area [25,26]. Open IOAM electromyogram recordings were assessed to identify the skeletal IOAM nerve intraoperatively before denervation. After grafting, the CMAP served as the functional test for the graft-induced IOAM regeneration. Positive CMAP tests analyze the type of neurotransmitter in axonal regeneration using Vecuronium: Glutamate (first motoneuron) in case of complete Ach-neuromuscular block or by competitive glutamatergic-cholinergic co-transmission (Figure 3).

### Statistical Analysis

**Figure 4:**
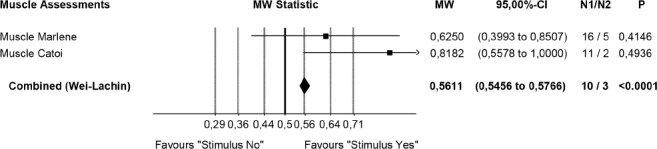
Synopsis of CERE medication effects on neuromodulation, neuroprotection, and neuro-recovery as observed in histology-morphology (blinded for 3 cohorts).

After peripheral nerve grafting, no single histopathological measure can capture the multidimensional nature of neuroplasticity. Therefore, we chose the highly efficient nonparametric Wei-Lachin procedure (a multivariate, correlation sensitive generalization of the Wilcoxon-Mann-Whitney test) with a multidimensional approach to evaluate the assessments statistically. We are well aware that the small sample sizes render parametric statistics sensitive to outliers (Figure 4) [28-30].

### Histologic morphologic analyses

Standard procedures were performed for all samples. Five-micron sections were cut from each block using a rotary microtome (RM 2125 RT, Leica), stained with hematoxylin and eosin (HE) and Masson-trichrome (MT), and examined under an Olympus BX51 microscope. Images were collected by an Olympus SP 350 digital camera (Olympus) and Olympus Cell B morphometric program for counting and measurements. 

Graft and graft-dependent secondary histology-morphology changes from T10 to L1 SC segments (approximately 5 mm) were identified in each case: description, measurement, and diagnoses within the SC, NG and surrounding area, e.g., type of regeneration, atrophy, vascularization, traumatic lesion, infection, and secondary lesions. The IOAM atrophy and regeneration of both sides were measured to be compared and statistically evaluated [31-39]. We developed our own scoring system for quantification by counting and measuring the area of the morphologic elements in at least 10 high-power fields (HPF = 400x) from serial slides. For more details, we refer to a procedure in combination with Western blot that we previously presented in another study [11].

Analysis and indirect immunofluorescence assessment of VGlut2 allowed for a more profound discussion on graft-induced central plasticity [1-4,11,40-43].

## Results

GB's microsurgical connection of CNS-PNS by PNG successfully replicated normal histology-morphology, demonstrating the correct positioning of the implanted sural NG within the right CST. The RCS protocol with additional postoperative IP medication and intensive care was adapted to a local veterinarian's ICU and to optimal housing, as both contributed to improved postoperative animal survival. Nevertheless, during three months of follow-up, sudden mortality occurred in 14 of 29 animals at different times without showing specific pathophysiological symptoms and signs or warning behavioral abnormalities; corpse findings in postmortem review were uneventful [1,11,19, 21-25]. One animal, control number 4, was lost intraoperatively when its autopsy on the operating table revealed accidental retroperitoneal perforation during the IOAM preparation to identify NEZ. This surgical mishap, however, led to an immediate modification of Brunelli's approach to an atraumatic microsocial preparation of the IOAM nerve for all following grafting operations, which were performed without any further surgical complications. Fifteen rats completed the full course RCT with euthanasia at the third operation (day 100) to harvest specimens of special interest for detailed histology-morphology analyses. When unblinded, the rats were revealed to be 3 controls (No. 5, 6, and 7), 7 NaCl (number 9, 12, 13, 18, 25, 26, and 29), and 5 CERE (No. 10, 11, 17, 24, and 27).

### Histology-morphology analysis

The total brain, SC, the graft, and right and left IOAM were analyzed in regard to graft-induced histology-morphology and pathology by CC. The specimens of IOAM from both sides were kept in Lübeck to assess graft-induced regeneration after denervation by comparative histometric muscle fiber analysis (ML).

### Electrophysiology [26,27]

To aid in identifying the IOAM nerve, the graft, and neuroregeneration of the grafted IOAM nerve were examined using intraoperative EMG (CAMP). Uncertainty about positive functional neuroregeneration in cases of negative EMG stimulation, when commonly related to technical problems, does not contradict positive regeneration, as positive regeneration was subsequently demonstrated by histometric measurement of grafted muscle fiber diameters. CAMP positive recordings were observed in 8 animals at the second operation, demonstrating the achieved extent of graft-induced muscle regeneration. Positive CAMP signals are necessary to analyze the type of neurotransmitter: 4 out of 8 CAMP+ rats received Vecuronium IV during artificial ventilation; two specimens showed the expected complete block of electrical stimulation, while two others showed a partial loss of CAMP+ amplitudes. The latter result strongly suggests mixed-type cholinergic/glutamatergic co-neurotransmission.

A total CAMP blockage is equated with glutamatergic (central?) reinnervation (Brunelli's paradigm) [2-4]. This first proof of graft-induced cholinergic/glutamatergic co-neurotransmission in GB's concept is not a contradiction of Brunelli's paradigm but additional valuable proof of induced central plasticity regarding its clinical potential for human SCI (Figure 4) [7,8,10,11].

### CERE pharmacological neuromodulation

**Figure 5:**
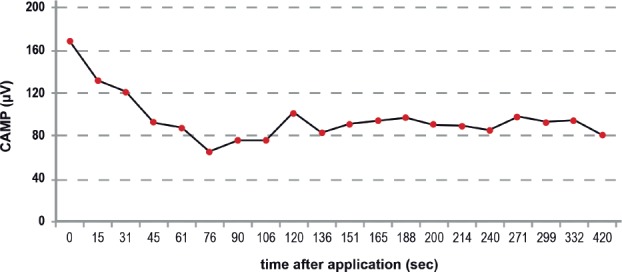
Electrical CAMP stimulation as proof of the actual neurotransmitter type. Positive CAMP stimulation during Vecuronium medication, although with reduced amplitudes, proves functional ACh-GLUT co-transmission neuroregeneration after IOAM (CERE No. 21), which was hypsometrically confirmed.

**Figure 6:**
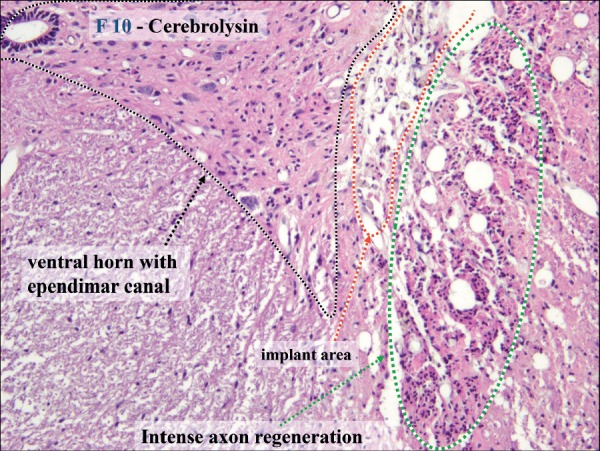
Histology of graft-induced neuroregeneration in the SC and PNG. In CERE specimen No. 10, intense polyfocal axonal regeneration was observed and was most pronounced next to the tip of NG and laminae 7-9; many Schwann cells were observed around new axons with outgrowth from the NG inside the SC as bands of Bünger, beneficial vascularization (HE 400x).

RCT experiments demonstrated the beneficial effect of postoperative CERE medication on functional neuroregeneration regarding larger IOAM fascicle diameters after atrophy compared with the two other cohorts when the statistical evaluation was unblinded. Neuroprotection by CERE was demonstrated by histology-morphology analysis, revealing less secondary scar formation around the tip of the graft and more intense axonal sprouting and vascularization next to the implanted graft in CST, inside the regenerated graft, and outside the SC [37-39] (Figure 5, Figure 6).

Postoperative temporary neurological deficit was more or less pronounced as the typical sequence of surgical manipulations of the SC during the grafting procedure in all 29 rats. Animals recovering from functional impairments showed good functional movement and neurobehavioral activity over time by demonstrating regular food intake and resumption of climbing, running, and playing [37,40,41,44]. Animals had free access to water and palette during the follow-up with regular assessments of body weight. Housing consisted of three animals in one cage with a room temperature of 21°C, air humidity of 50%, and 12 hours of light-dark. All measurements were taken and reported in the animal's protocol housing to minimize suffering from pain or neurobehavioral deficits.

The histology – morphology revealed a final CNS-PNS connection by describing and explaining in detail all aspects of interest in this research, e.g., individual microsurgical free-hand positioning of NG within and next to the SC and of the graft itself inside and outside of the SC as well as its adaptation at the IOAM-NEZ, together with all diagnosed primary and secondary surgical lesions related to the given RCT design [11,31-39]. Demonstration of the graft and its positioning within the right lateral corticospinal tract was possible. Suspected morphological variety of Brunelli's free-hand microsurgical technique of putting the NG tip into the targeted lateral cortical-rubrospinal tract was possible in all preserved specimens (Figure 7. AB). Secondary cell death, hemorrhages, scar formation (grade 1 to 3), atrophy, and graft displacement could be analyzed and diagnosed in detail. New aspects of graft-induced polyfocal axonal regeneration were discovered and were related to different SC Rexed areas that were previously interpreted in the first report [11]. Therefore, unblinded EMG-negative cases could be related to the Rexed laminae 3-5, while in all EMG-positive cases (but also in two negative ones), the graft was located in laminae 7-10 on the graft side [11]. Angiogenesis appeared more intense in EMG-positive cases. Gitter cells were identified in all cases around and inside the SNG. Remarkably less scarring was observed in the CERE cohort, while three different grades of fibrosis intensity in SC were connected to the graft but were not statistically correlated in this research (Figure 6, Figure 7) [18,26-39].

**Figure 7A:**
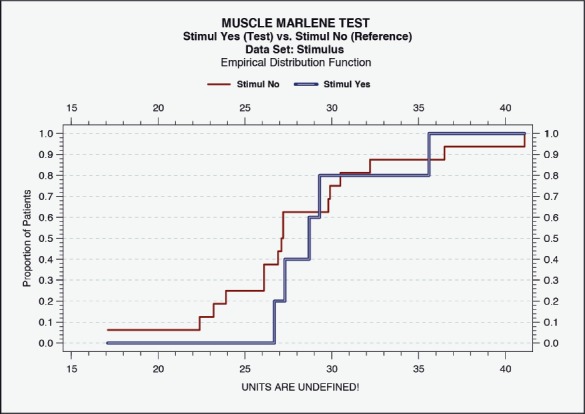
Histometric evidence of positive IOAM regeneration by a count of muscle fiber diameter after atrophy. After CERE treatment, increased fiber diameters were more pronounced (calculation by ML). Empirical Distribution Function. Muscle stimulus “Yes” (test) versus “No” (reference). Data Set: Stimulus; Empirical Distribution Function.

**Figure 7B:**
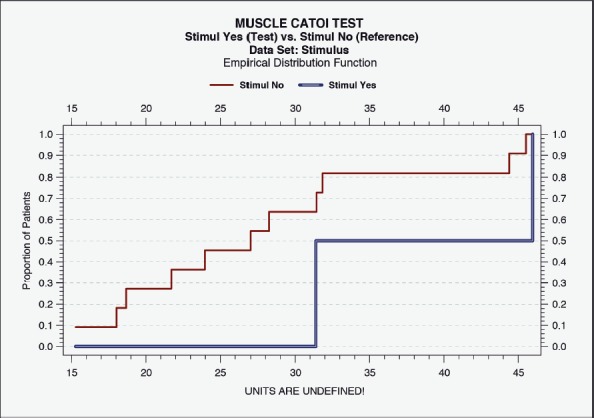
Correlation of IOAM fiber diameter with CAMP+ stimulation. All specimens were analyzed by CC. Empirical Distribution Function, not a correlation analysis. Muscle stimulus “Yes” (test) versus “No” (reference). Data Set: Stimulus.

### Statistical analysis

**Figures 8:**
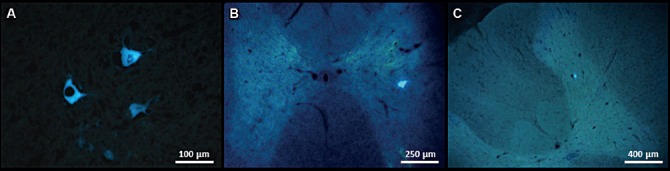
AB New glutamatergic axons and axonal terminals in PNG outside SC and IOAM (cross-section; magnification 630x). **A.** Outside the SC. PNG cross-sections: axons with green fluorescence FITC (white arrow), Swann cell nuclei stained in red (empty arrows) (FITC-conjugated goat anti-rabbit IgF + rabbit anti VGLUT2, DRAQ5; CERE, No. 10). **B.** IOAM cross-section: neuroregeneration with glutamatergic axons and axonal terminals with green fluorescence FITC (white arrows), Schwann cell nuclei stained red (DRAQ5; NaCl No. 13).

Statistical evaluation (Wei-Lachin procedure) of the specimens for IOAM fiber diameters were analyzed and identified by the histometric assessment. PNG-induced functional neuroregeneration was evident in both independently assessed measurements (ML and CC) (Figure 7, Figure 8) [11,17,18,42-44]. Although statistical evaluation revealed clear evidence for CERE neuromodulation based on a larger IOAM fiber, the limited number of animals provided large variations within controlled histometric data analysis of fiber thickness after regeneration of IOAM following atrophy after denervation compared with the intact ones. However, in this statistical evaluation of muscle assessments by multidimensional histology, the Wei-Lachin procedure showed a clear tendency for CERE neuroprotection stimulus "Yes" vs. stimulus "No," with effect sizes and two-sided 95% CI (Figure 7) [16-18, 28-30]. This proof of regenerated IOAM morphology is consistent with prior open intraoperative EMG results (CAMP+ records) at three months postoperatively (second operation). The EMG-positive cases, in general, exhibited a larger average muscle fiber diameter when reaching 75% of the contralateral intact muscle fiber diameter. The histometric morphology of muscle fibers can definitively confirm IOAM regeneration, particularly when the intraoperatively limited meaningfulness of open intraoperative EMG recording limits the CAMP+ related allocation of animals for Vecuronium medication in RCS. However, due to the small sample sizes, the test results should be interpreted with care.

Indirect immunofluorescence of VGluT2 was used in two CAMP+ cases to diagnose graft-induced neurotransmitters in PNG and IOAM specimens (Figure 8. AB). To date, three different vesicular glutamate transporters, VGluT1, VGluT2, and VGluT3, are considered the most specific markers for neurons that use glutamate as a neurotransmitter [40]. In specimens number 10 (CERE) and number 13 (NaCl), all three VGluTs were identified within the SC (in the perikaryon (soma) and processes of specific FB+ neurons) in different Rexed areas at different levels, both anterior (thoracic segments 9-11) and posterior (lumbar segments 1-3) to the site of SNG implantation (segments adjacent to SNG implantation were used before in routine histology). VGluT2 within IOAM axonal terminals was only demonstrated in the one specimen (number 13 NaCl at the second operation) - Figure 8. AB [40].

Replication of GB's concept shows the capacity of spinal VGlutT2 neurons to regenerate PNG and to produce terminals that reinnervated the IOAM skeletal muscle (GB paradigm) that correlates with the Western blot analyses of coexisting acetylcholine and glutamate neurotransmitters shown both within the SC and the regenerated IOAM nerve. The co-neurotransmission is in contrast to GB's paradigm, and we did not reveal any retrograde FB+ tracer fluorescence within the brainstem or brain. When our intraoperative FB+ application (during the second operation) was different from GB's technique, the application was consistent with the labeling techniques used by M. Schwab and coworkers [11,44]. This discrepancy regarding the neurotransmission by the first and/or second motoneurons requires additional experimental proof using Fast Blue labeling [ 2,4,44]. An fMRI case reports a clinically successful follow-up after GB's CNS-PNS grafting concept of hip muscles after paraplegia due to traumatic SCI - guillotine lesion at the T8-T9 level after a car accident in the year 2000, before the patient turned 30 years old. In 2018, the patient was still able to walk and climb steps using tetrapod sticks. She reported having "a personal quality of life, which is more than satisfying" until the present, 2018 [45-49,60,61]. In 2015, the patient submitted to the first fMRI three Tesla neuro-radiological diagnostic assessment of her brain function at the SAN RAFFAELE Institute in Milano [46,47]; the scan was obtained after her daily intensive physical training and after she experienced the first active muscle activation of her lower limbs, which occurred at two years postoperatively. Daily voluntary standing and ambulation using tetrapod sticks and social activity became routine for the patient approximately two years later.

Irrespective of GB's promising scientific reports, the paradigm for partial functional restoration remains controversial without additional external proof. Every novel medical concept in restorative surgery, such as here for spinal cord repair, should be first replicated, validated, and discussed in light of GB's report. Clinical fMRI performance of client G during her brain functional diagnostic 15 yers after GB's grafting procedure in Italy "has upset all the doctors present because they saw a patient with a broken medulla and a client that pedaled, extended knees and abducted her hips in following the radiologist's commands." The car accident occurred at the time of her wedding, and she got divorced soon after her SCI and had to learn to manage life on her own. Over the years, she has been in personal, friendly contact with GB and his wife, Luisa Monini Brunelli, Professor of Orthopedics, who assisted in the grafting operation in 2000. The patient, unfortunately, has refused any state of the art professional neurological/neurophysiological/neuro-radiological follow-up examination and external medical audit. However, the assessment of the surgical results by an objective third party is mandatory to objectively evaluate the first experimental of GB's microsurgical concept regarding neuroregeneration in traumatic thoracic paraplegia (the operation was performed by GB and KvW). Her detailed fMRI evaluation by a third party at the Department of Radiology, Münster UKM, will be published in the near future in conjunction with her first professional external neurological expert assessment. This paper focuses on understanding the special role of the rubrospinal tract in the functional regeneration of denervated muscles after PNS-CNS grafting. Second, the role of intensive neurorehabilitation for the functional outcome is stressed to induce regeneration and to maintain partial restoration of skeletal muscle groups after successful grafting when translated into human SCI repair. Aspects of subjective wellbeing and personal HRQOL following SCI are still underestimated [48,49,53,55,56,60,61].

Prof. Andrea Falini from Milano wrote in her original medical report of GB's first fMRI when analyzing her voluntary muscle activities by obeying different simple motor tasks both physically and/or imaginary:" Having given the patient orders during her only fMRI studies to do certain movements, for example, tasks such as abduction or extension of hip muscles and then to "think" only. In conclusion, the preliminary report of data analysis, which we tried to carry out by automatically identifying the membership of the activation clusters to cortical areas defined according to atlases implemented in the processing software. We had the impression that the motor stimulus of the right lower limb produced a rather typical activation in the left paracentral lobule and in the premotor area, with some clusters in the right frontal operculum (area 44?), and perhaps temporal left, which deserve attention." Another external audit of the total examination following our strict criteria and performed by an expert in functional neuroradiology at Münster UKM, Germany, is forthcoming and will be discussed in more detail in a future medical report that will focus on brain reorganization after SCI and restoration of muscle functioning [46,47].

## Discussion

Numerous surgical, medical, and biochemical strategies that have been based on experimental trials are recommended and used clinically in human SCI rehabilitation to foster the restoration of voluntary muscle function and beneficial personal HRQOL after traumatic spinal cord disruption with permanent paraplegia [11,18,48,53,60, 61]. Muscle atrophy occurs rapidly after denervation, while graft-induced restoration of function and muscle mass by reinnervation is a slow process, as it occurs in healthy muscles. Axonal regeneration is not synonymous with complete functional restoration, and complete or incomplete functional recovery can be progressive.

Current clinical treatment strategies are targeted at promoting axon regeneration and outgrowth beyond the scar formation in SC avulsion. The strategies are all based on the preserved central plasticity of a functioning upper SC. Neuroplasticity is the ability of the nervous system to respond to intrinsic or extrinsic stimuli by building and reorganizing its structure, function, and connections [9,10,11,41-43,45,51,53]. Notwithstanding the evidence that spinal axons grow into the PNG [1], the number and thickness of the axons and their ion channel expression patterns may not be identical to those necessary for their characteristic signal propagation. This fact may explain the limited functional restoration as it was assessed in only 8 of our animals by CAMP+ stimulation after three months. The regeneration of IOAM is also of special importance. This is the first observation of the coexistence of graft-induced cholinergic (peripheral) and glutamatergic (central) neurotransmission in the reinnervated IOAM (immunoblotting). Then, FB+ neurons were confirmed in numerous laminae of the SC gray matter. However, these considerations do not contradict GB's and coworkers' views on GB's paradigm but instead support the actual scientific hypothesis that CNS plasticity mediates PNG regeneration by different types of spinal neurons (motor, sensory, and interneuron) [5,11 36-40,46-51]. The coexistence of both neurotransmitter types in regenerated skeletal muscles is not surprising. Filli and coworkers noted from their experimental observations that: "severed reticulospinal fibers, which are part of the phylogenetically oldest motor command system, spontaneously arborize and form contacts onto a plastic propriospinal relay, thereby bypassing the lesion" [44]. Rearrangements were accompanied by a substantial locomotor recovery that was based on the regenerative capacity of the rubrospinal tract and lower motor neurons. These new observations open new avenues for clinicians for restorative microsurgery in select spinal cord lesions, as intended by GB's concept in clinical practice [44]. To date, numerous literature reports have analyzed experimental research concepts for animals or human SC treatment, e.g., aspects of the enhancement of endogenously occurring spontaneous remyelination, the role of adaptive immune responses in remyelination and immunoregulatory potential of the glial scar and genetic manipulation, immunomodulation, manipulation of the glial scar and cell transplantation, and nanotechnology and tissue engineering products, suggesting additional opportunities, reviewed in detail by Papastefanski and Matas [53]. The regeneration and restoration of some targeted skeletal muscles using an implanted or functional electrical exoskeleton of SC remain experimental and limited to a few laboratories [54]. Scientific replication and long-term follow up are mandatory to translate singular beneficial experimental results into clinical application for beneficial SCI restorative management to be ethically approved [9,10,48, 52,53,55]. When translating animal models into human clinical management, the fully informed and enlightened layman, the client, should be in contact with the expert reconstructive plastic microsurgeon first to learn in detail about the chances and risks of the responsible person. The clinician should make his decision based only on the SCI, patient's personal wishes, energy, and social competence, as demonstrated by Mrs. G's follow up rehabilitation and HRQOL. Because GB's successful but limited concept has not been considered by other rehabilitation physicians in cases of SCI paraplegics with damage at the level of T6-T8, numerous suitable and qualified paraplegics have missed their chance to recover upright ambulation by their will and energy [60-63]. In addition, despite several evidence-based scientific reports, CERE medication is still not recommended as an additional therapy to support early posttraumatic SCI management and neuromodulation following restorative microsurgery and neurorehabilitation, at least in a first clinical prospective RCT. Based on our limited experimental research in the rat model, we would recommend pharmacological neuromodulation by CERE, a neuropeptide preparation that mimics the action of endogenous neurotrophic factors for brain and SC protection and repair. Natural neurotrophic factors are key determinants involved in CNS protection and recovery, with the ability to switch DNA programs. Restorative processes, such as axonal regeneration, neuronal plasticity, and neurogenesis, are essential for the future recovery of conditions such as spinal cord or traumatic injury [16-18]. Masliah and Dies-Tejedor describe the neuroprotective effects of CERE on experimentally-induced traumatic spinal cord injury. In this paper, the authors assert that CERE prevents apoptosis of lesioned motoneurons and promotes functional recovery [15]. Second, Haninec et al. found that CERE treatment for motor neuron maintenance and survival resulted in the functional reinnervation of the nerve stump in a C5 ventral root avulsion experimental rat model. In this case, morphometric measurements confirmed that treatment greatly protected the motor neurons against cell death. Moreover, CERE had superior results for functional recovery and cell-death prevention compared to brain-derived neurotrophic factor (BDNF) [18]. Consistent with the previously described literature findings, the limited beneficial results of our RCS, including CERE neuromodulation, show decreased fibrosis and intense angiogenesis in and around the NG graft in subjects that received acute CERE administration. Additionally, the diameters of IOMA fibers appeared marginally larger compared to controls, indicating a clear tendency toward positive pharmacological neuromodulation and neuroregeneration, although statistical inference was limited due to the small number of animals assessed. Our results indicate a strong potential correlation between CERE-attributed neuroprotection and neuro-recovery and positive electrical stimulation, as a measure of improved surgical outcome. Further study with improved design and samples is therefore warranted to establish causality for this mechanism. In contrast to these observations, a cross-sectional quasi-experimental trial by Keyilhoff et al. on the effects of CERE on motor-neuron-like NSC-34 cells only reports isolated positive effects of CERE on injured motor neurons at the spinal cord level [59] The actual experimental study presented here suggests that conditions for safe clinical administration in spinal cord injuries must be explored. Nevertheless, we assert that this study was performed in an isolated environment, potentially confounding positive results. We, therefore, reassert our previous recommendation for future studies on this topic. Regarding the safety of administration, in two studies that explored the coadministration of CERE with iron oxide magnetic nanoparticles (IOMNPs), CERE mitigated IOMNP effects on SCI, produced important neuroprotection, and reduced the leakage of plasma proteins. CERE also decreased the overall number of neurons that were injured. These studies also indicate the safety of CERE in preventing CNS pathology after an SCI and IOMNP administration [57-59].

## Conclusions

In accordance with Dopkin's demand and the statement of Papastefanski and Matas, new strategies are needed in neuroscience to improve the transparency of experimental descriptions by uniform reporting. This first replication of Brunelli's grafting procedure in a rat model provides new insight into graft-induced central neuroplasticity and neuroregeneration of denervated skeletal muscles, and the cortical-rubrospinal tract has shown to be crucial in neuroregeneration, which was first discussed as the fundamental structure by GB in his CNS-PNS concept and is now also confirmed in stroke rehabilitation. The actual evidence of acetylcholine/glutamatergic co-transmission in the grafted muscle must be replicated in a more extensive series, including pharmacological neuromodulation and additional behavioral outcome assessments by extended follow-up controls. SCI paraplegics may receive GB's clinical restorative microsurgery when presenting CNS-PNS graft-induced glutamatergic/cholinergic co-transmission, which will open a new discussion of the targeted aspects of neuroregeneration and neurorehabilitation. Eighteen years of follow-up and actual fMRI examination after GB's grafting of incomplete thoracic SCI provide evidence that the client was able to voluntarily stand and walk using tripod sticks with good personal physical-medical and mental-social quality of life (HRQOL). However, this type of experimental surgery requires the client's explicit consent with regular neurological/neuropsychological and social follow-up examination and an external audit, including fMRI, at least four years after the intervention. Over the last two decades, we have learned from our numerous clients in restorative microsurgery and in scientific discussions with other specialists and rehab doctors worldwide that these recommendations are best for the paraplegic patient's neurorehabilitation.

## Conflict of Interest

The authors confirm that there are no conflicts of interest.
